# Use of clustering analysis in randomized controlled trials in orthopaedic surgery

**DOI:** 10.1186/s12874-015-0006-1

**Published:** 2015-03-08

**Authors:** Hanna Oltean, Joel J Gagnier

**Affiliations:** Washington State Department of Health, Seattle, WA USA; Department of Orthopaedic Surgery, University of Michigan Health System, MedSport, Domino’s Farms, 24 Frank Lloyd Wright Drive, Ann Arbor, MI 48105 USA; Department of Epidemiology, School of Public Health, University of Michigan, Ann Arbor, MI USA

**Keywords:** Randomized controlled trials, Clustering analysis, Orthopaedic surgery, Statistical analysis

## Abstract

**Background:**

The effects of clustering in randomized controlled trials (RCTs) and the resulting potential violation of assumptions of independence are now well recognized. When patients in a single study are treated by several therapists, there is good reason to suspect that the variation in outcome will be smaller for patients treated in the same group than for patients treated in different groups. This potential correlation of outcomes results in a loss of independence of observations. The purpose of this study is to examine the current use of clustering analysis in RCTs published in the top five journals of orthopaedic surgery.

**Methods:**

RCTs published from 2006 to 2010 in the top five journals of orthopaedic surgery, as determined by 5-year impact factor, that included multiple therapists and/or centers were included. Identified articles were assessed for accounting for the effects of clustering of therapists and/or centers in randomization or analysis. Logistic regression used both univariate and multivariate models, with use of clustering analysis as the outcome. Multivariate models were constructed using stepwise deletion. An alpha level of 0.10 was considered significant.

**Results:**

A total of 271 articles classified as RCTs were identified from the five journals included in the study. Thirty-two articles were excluded due to inclusion of nonhuman subjects. Of the remaining 239 articles, 186 were found to include multiple centers and/or therapists. The prevalence of use of clustering analysis was 21.5%. Fewer than half of the studies reported inclusion of a statistician, epidemiologist or clinical trials methodologist on the team. In multivariate modeling, adjusting for clustering was associated with a 6.7 times higher odds of inclusion of any type of specialist on the team (*P* = 0.08). Likewise, trials that accounted for clustering had 3.3 times the odds of including an epidemiologist/clinical trials methodologist than those that did not account for clustering (*P* = 0.04).

**Conclusions:**

Including specialists on a study team, especially an epidemiologist or clinical trials methodologist, appears to be important in the decision to account for clustering in RCT reporting. The use of clustering analysis remains an important piece of unbiased reporting, and accounting for clustering in RCTs should be a standard reporting practice.

## Background

The effects of clustering in randomized controlled trials (RCTs) and the resulting potential violation of assumptions of independence are now well recognized [1,2]. For example, when patients in a single study are treated by several therapists, there may be reason to suspect that the variation in outcome will be smaller between patients treated by the same clinician than between patients treated by different clinicians [2-4]. Clustering effects may arise when there is a potential for correlation of outcomes among patients in similar groups, which can result in a loss of independence of observations. Analyses taking into account correlation among patients may be especially important for studies involving treatment and care that depend on substantial skill or training; however, differential therapist effects may also arise due to personality differences, personal experience, or infrastructure [1,2,5,6]. Clustering may also occur by location (e.g., in a multicenter trial or inpatient vs. outpatient facilities) or in a study that randomizes by cluster (a cluster randomized trial), in which those delivering the intervention (e.g., surgeons) represent different clusters. On occasion, differences between clusters may be an outcome of interest to researchers; however, the outcome in RCTs is typically a measure of effectiveness of some intervention, irrespective of any clusters. That is, if the effectiveness of an intervention is influenced by some cluster (e.g., the healthcare provider delivering treatment) and if this is not measured or accounted for in the study, this introduces a source of bias into any outcomes measured [6]. These potential sources of bias (e.g., center cluster or therapist cluster) have implications for outcome effect magnitudes and directions, and thus should be recognized and treated accordingly. The design and analysis of RCTs should account for the possible heterogeneity in cluster size and intracluster correlation to appropriately analyze results [2].

The majority of statistical analyses used in RCTs are based on the assumption that observed outcomes on different patients are independent [7]. Independence of observations is a basic assumption of many widely used statistical tests, including t-tests and generalized linear modeling. Between-cluster variation may therefore lead to a loss of precision and reduced power when estimating treatment effects [2,6]. Clustering therefore has implications for the required sample size of an RCT; the impact depends on the study design and analysis used [1]. The magnitude of the effect additionally depends on cluster size and intracluster correlation coefficients (ICCs) [2]. The extent to which the treatment effect varies across clusters can have a major impact on the interpretation of a trial’s results; however, there is often not enough information to obtain a precise estimate of the clustering effect, since most trials are not powered to detect this variability [7]. The magnitude of clustering may depend on cluster type, setting, and type of outcome, as well as time since receiving the intervention [1]. Therefore, if clustering is believed *a priori* to be a realistic possibility, it is important to account for it in analysis to appropriately interpret the treatment effect [7].

These effects are illustrated in a study by Lee and Thompson [7], in which two published trials were re-analyzed using an analysis method that accounted for the effects of clustering, which was not used in the original publication. They found that if potential clustering is ignored, uncertainty may be underestimated, producing too extreme p values and even altering the results of a trial [7]. In their first re-analysis, the authors looked at a trial assessing the effectiveness of teleconsultations performed by 20 consultants. The original study analyzed the observations as independent and concluded that the treatment was significantly more effective than the control. In a re-analysis of the study data using a random effects model, Lee and Thompson [7] found that clustering by consultant was significant. When this clustering was controlled for in the model, the resulting odds ratio became nonsignificant, therefore altering the results of the trial. In the re-analysis of a second study, the results of an exercise class delivered by 21 physiotherapists were called into question when it was determined that the standard error in a model controlling for clustering was larger than originally determined. This suggested a wide variation in treatment effect and again alters the interpretation of the study results.

In a second study that re-analyzed the data of two clinical trials to account for clustering, Roberts and Roberts [2] again found that the standard errors of the treatment effects markedly increased. A study by Cook et al. [1], analyzing ICCs for 198 outcomes across 10 multicenter surgical trials, demonstrated clustering effects at both the center and surgeon level and concluded that clustering of outcome is more of an issue than has been previously acknowledged. These examples demonstrate the dramatic effect that clustering may have and the mistaken conclusions that can be drawn if it is ignored in the analyses.

In one study specifically assessing a large orthopaedics surgical trial, Biau et al. [8] found provider effects to be highly significant in re-analysis. These provider effects were found to be more significant in highly specialized fields, such as orthopaedics, in contrast to general surgery [8]. Using volume of patients seen per surgeon as a proxy for surgeon experience, higher surgeon experience was shown to correlate with better patient outcomes [8]. This study therefore suggests that controlling for clustering effects is especially important in studies that involve highly skilled therapists.

Clustering in randomized clinical trials can be dealt with in many ways. Several methods of accounting for clustering are widely recognized: randomizing patients within each cluster (e.g., to the treatment provider), cluster-level analysis, fixed-effects models, random effects models, or generalized estimating equations [6,9].

Despite multiple studies demonstrating the importance of clustering analysis and available methodological and statistical approaches for handling it, accounting for clustering is not routine in the analysis of published RCTs [10]. Based on findings in the general literature [4,7,10], we hypothesized that the prevalence of the use of clustering analysis reported in the orthopaedic literature would be low. Studies in the field of orthopaedics often involve highly skilled therapists and therefore have great potential to be affected by clustering [8]. The primary objective of the present study was to determine the prevalence of reporting of the use of clustering analysis in RCTs published in the top five orthopaedic journals between 2006 and 2010. A secondary objective was to identify factors predicting the use or neglect of use of clustering analysis in the RCTs included in this study.

## Methods

### Identification of articles

We identified the top five journals with the highest 5-year impact factor in the area of orthopaedics as listed in the 2010 ISI Web of Knowledge Journal Citation Reports [11]. Journals included were: *American Journal of Sports Medicine* (AJSM), *Journal of Bone and Joint Surgery* (JBJS), *Journal of Orthopaedic Research* (JOR), *Osteoarthritis and Cartilage* (OC), and *The Spine Journal* (SJ). All articles in all issues of these journals published between 2006 and 2010 were hand-searched. Inclusion criteria for articles were: randomized allocation of participants to two or more groups, inclusion of human subjects, and inclusion of a potential grouping variable (e.g., multiple therapists or treatment centers). Articles were excluded from analysis if they included nonhuman subjects, if they were conducted in a single center by a single therapist, or if they did not report enough information to determine inclusion. A single individual assessed all articles for inclusion (HO) and a random proportion, approximately 10%, were checked by a second individual (JG) to ensure eligibility. These two individuals met to discuss any disagreements, which were resolved by discussion.

### Data extraction

Articles meeting the inclusion criteria were then searched for the following data: reporting of the number of therapists delivering treatment, number of centers used in the study, whether or not multiple therapists or centers were accounted for in randomization or statistical analysis, year of publication, the impact factor of the journal in the publication year, whether the study team included a statistician, whether the study team included an epidemiologist or clinical trials methodologist, the sample size of the study, and whether the primary outcome (defined as the outcome described as primary, to which the study was powered, or the first outcome reported) of the study was categorically positive, neutral, or negative. A statistician was defined as any person with a graduate degree in statistics or biostatistics. An epidemiologist/clinical trials methodologist was likewise defined as any person with a graduate degree in epidemiology, public health, or clinical research. The corresponding author was contacted via e-mail and asked if either specialist was included on the study team, if this information was not clear from the published report. The outcome of the study was defined as positive if the study findings supported the *a priori* hypothesis, defined as negative if the primary outcome was in the opposite direction hypothesized, and neutral if no significant effect was demonstrated.

The outcome measure of interest was accounting for clustering by therapist, accounting for clustering by center, and accounting for any type of clustering, either in randomization or analysis. All data were extracted by one individual with expertise in epidemiology and biostatistics.

### Statistical analysis

Data were compiled in Excel (Microsoft, Redmond, Washington) spreadsheets and imported into SAS, version 9.3, statistical software (SAS Institute Inc., Cary, North Carolina) for statistical analysis. Frequency measures were computed for all data. Logistic regression was conducted using both univariable and multivariable models. Univariable logistic regression was performed for each predictor variable on each outcome variable, with no adjustments. Multivariable models were constructed using stepwise deletion, with deletion of the variable with the highest p value in each case. All variables were checked for collinearity before inclusion in multivariable models, and collinear variables were tested separately. Clustering effects by journal were checked using generalized estimating equations (GEE). Odds ratios and 95% confidence intervals were produced in all analyses. An alpha level of 0.10 was considered significant for all tests [12]. Confidence intervals reported are at the 95% level.

## Results

**Figure 1:**
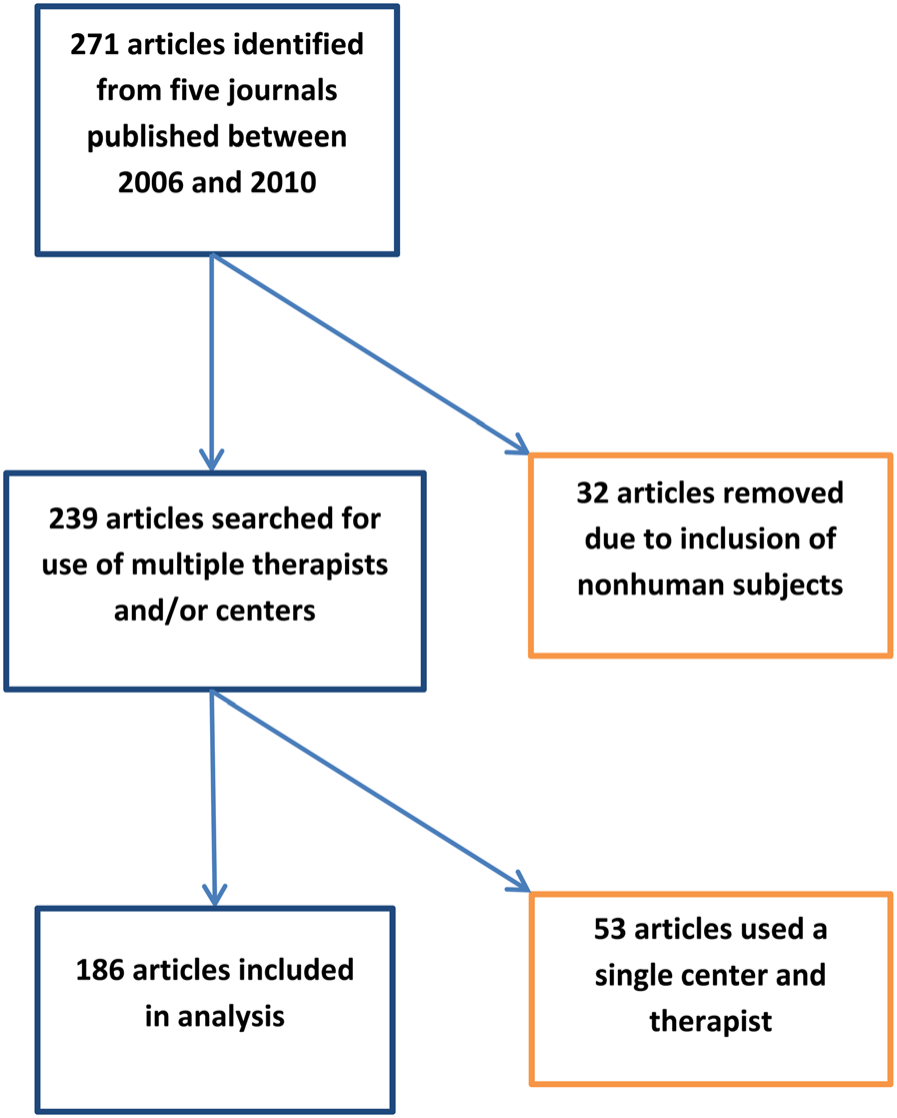
**Flow chart of article inclusion.**

A total of 271 articles classified as RCTs were identified from the five journals included in the study (Figure 1). Thirty-two articles were excluded due to inclusion of nonhuman subjects. The remaining 239 articles were screened for multiple therapists and/or centers. Of these, 186 were found to include multiple centers and/or therapists and were used in our analysis. Eighty-seven studies (46.7%) included multiple centers, and 145 studies (78.0%) included multiple therapists (Table 1). Of the 186 articles included, 40 (21.5%) accounted for clustering on some level, either in randomization or statistical analysis (see Table 2). Ninety-one studies (48.9%) reported inclusion of a statistician on their study team, and 83 (44.6%) reported inclusion of an epidemiologist or clinical trials methodologist. However, a description of the statistician training was missing in 60 of the studies, and data on epidemiologist/clinical trials methodologist were missing in 67 of the studies. Studies reporting a positive outcome numbered 94 (50.5%), while negative outcomes comprised only 6 of the studies (3.2%), and neutral outcomes were reported in 86 (46.2%). All but one article reported a sample size; the average sample size among included articles was 200.5 (95% CI, 150.8 to 250.2), with a range of 16–2483 study participants.

**Table 1:** **Characteristics of articles included in analysis (N = 186)**

	**N**	**%**
Multiple centers	87	46.7
Multiple therapists	145	78.0
Reported any clustering analysis	40	21.5
Reported inclusion of a statistician on study team	91	48.9
Report inclusion of a clinical trials methodologist or epidemiologist on study team	83	44.6
Reported inclusion of either specialist	109	58.6
Reported inclusion of both specialists	65	34.9
Reported positive outcome	94	50.5
Specified null hypothesis but reported positive outcome	31	16.7
Reported negative outcome	6	3.2
Reported neutral outcome	86	46.2
Sample size (mean, SD)	185	200.5, 344.8

**Table 2:** Methods used to account for clustering (N = 40*)

**Method**	**N (%)**
Stratified randomization	23 (57.5)
Cluster randomization	7 (17.5)
GEE or controlled variable	9 (22.5)
Stratified analysis	8 (20.0)
Other	1 (2.5)

*6 studies used two or more methods to account for clustering.

Univariable logistic regression of predictors on the use of any type of accounting for clustering is shown in Table 3, which compares articles containing these predictors to those that do not. Three predictors were found to have a significant association with accounting for clustering in univariable modeling: sample size, inclusion of an epidemiologist/clinical trials methodologist, and inclusion of any type of specialist. Trials that accounted for clustering had 3.6 times greater odds of including an epidemiologist/clinical trials methodologist on their author list or research team than those that did not account for clustering (95% CI, 1.2-11.4, *P* = 0.03). Additionally, sample size appears to have a significant association with accounting for clustering in randomization or analysis; however, the effect size is very small (odds ratio (OR) = **>** 1.0, 95% CI, 1.0-1.0, *P* = 0.04). The inclusion of any type of specialist (biostatistician and/or epidemiologist) yielded a very high odds ratio, with studies accounting for clustering being 6.4 times more likely to include a specialist (95% CI, 0.8-50.0, *P* = 0.08). All other variables tested in univariable analysis had relatively small effect sizes with insignificant p values (Table 3). No significant differences were seen between individual journals in their likelihood to account for clustering (data not shown), nor were any significant changes seen when adjusting for clustering by journal using GEEs.

**Table 3:** **Logistic regression of use of any clustering analysis by predictors (N = 186)**

	**OR**	**95% CI (** ***P*** **value)**
Any specialist^(119)^	6.4	0.8-50.0 (0.08)
Both specialists^(119)^	2.0	0.8-4.6 (0.13)
Biostatistician^(126)^	1.5	0.6-3.9 (0.39)
Epidemiologist/Methodologist^(119)^	3.6	1.2-11.4 (0.03)
Sample size^(185)^	1.0	1.0-1.0 (0.04)
Journal^(186)^	1.2	0.9-1.6 (0.25)
Impact factor^(186)^	1.4	0.8-2.6 (0.23)
Outcome^(186)^	1.0	0.5-1.9 (0.98)
Outcome2*^(186)^	1.0	0.7-1.6 (0.93)

*Outcome 2 separates positive outcomes into those that specified the null hypothesis and those that did not.

Univariable logistic regression of predictors on the use of clustering analysis to account for multiple centers is shown in Table 4. The odds ratios for inclusion of any type of specialist on the study team and inclusion of a biostatistician on the study team were 2.3 and 1.9, respectively; however, the 95% confidence intervals were large and did not indicate significance. Other included predictors had small effect estimates and none were found to have a significant association with the outcome variable at alpha = 0.05.

**Table 4:** **Logistic regression of use of clustering analysis for multiple centers by predictors (N = 87)**

	**OR**	**95% CI (** ***P*** **value)**
Any specialist^(64)^	2.3	0.2-20.7 (0.47)
Both specialists^(58)^	1.4	0.4-4.7 (0.60)
Biostatistician^(64)^	1.8	0.5-7.9 (0.35)
Epidemiologist/Methodologist^(58)^	1.1	0.3-4.9 (0.87)
Sample size^(86)^	1.0	1.0-1.0 (0.94)
Journal^(87)^	1.4	1.0-2.0 (0.07)
Impact factor^(87)^	1.4	0.6-2.9 (0.41)
Outcome^(87)^	1.3	0.6-3.0 (0.51)
Outcome2^(87)^	1.3	0.7-2.4 (0.38)

Table 5 shows univariable logistic regression of predictors on the use of clustering analysis to account for multiple therapists. Again, most of the included predictors had small effect estimates with wide confidence intervals; the exception was inclusion of an epidemiologist/clinical trials methodologist on the study team, with an OR of 5.7. This effect estimate does have a wide confidence interval, which again crosses the line of no effect, 0.7-45.9.

**Table 5:** **Logistic regression of use of clustering analysis for multiple therapists by predictors (N = 145)**

	**OR**	**95% CI (** ***P*** **value)**
Any specialist^(96)^	-	undefined
Both specialists^(96)^	1.3	0.4-4.4 (0.63)
Biostatistician^(101)^	0.9	0.3-3.1 (0.86)
Epidemiologist/Methodologist^(93)^	5.7	0.7-45.9 (0.10)
Sample size^(143)^	1.0	1.0-1.0 (0.21)
Journal^(145)^	1.0	0.6-1.5 (0.85)
Impact factor^(145)^	1.3	0.5-3.1 (0.57)
Outcome^(145)^	0.7	0.3-1.7 (0.41)
Outcome2^(145)^	0.7	0.4-1.4 (0.35)

Multivariable models are depicted in Table 6 and Table 7. An initial full model, Table 5, containing all variables was reduced to three separate models, Table 6, due to the significantly high collinearity of the variables ‘including any type of specialist’, ‘including a biostatistician’, and ‘including an epidemiologist/clinical trials methodologist’. Any variables with undefined ORs were excluded. The first model contains inclusion of an epidemiologist/clinical trials methodologist and is adjusted for sample size, journals, impact factor, and outcome. This model shows a statistically significant association, after controlling for sample size, journal, impact factor and outcome, between any accounting for clustering and inclusion of an epidemiologist/clinical trials methodologist, OR = 3.3 (95% CI, 1.0-10.4, *P* = 0.04) and separately for inclusion any type of specialist OR = 6.7 (95% CI, 0.8-57.3, *P* = 0.08) but not for inclusion of a biostatistician, OR = 1.5 (95% CI, 0.6-4.1, *P* = 0.40). Sample size was again seen to be significant in several of these models; however, it consistently had a very small effect estimate (OR = 1.0). The results of the stepwise deletion are also shown with only epidemiologist/methodologist and any specialist remaining in the model.

**Table 6:** **Multivariable analysis of any use of clustering analysis, stepwise deletion**

	**Model 1 OR(CI),** ***P*** **value**	**Model 2 OR(CI),** ***P*** **value**	**Model 3 OR(CI),** ***P*** **value**	**Model 4 OR(CI),** ***P*** **value**	**Model 5 OR(CI),** ***P*** **value**	**Model 6 OR(CI),** ***P*** **value**
Any specialist	3.9(0.3-60.3), 0.32	3.5(0.3-40.8), 0.32	3.5(0.3-40.8), 0.32	3.6(0.3-42.5), 0.31	3.7(0.3-42.9), 0.30	
Biostatistician	0.9(0.3-2.8), 0.84					
Epidemiologist/Methodologist	2.0(0.5-8.1), 0.32	2.1(0.5-8.0), 0.29	2.1(0.5-8.1), 0.28	2.1(0.5-7.8), 0.29	2.0(0.5-7.7), 0.30	3.3(1.0-10.4), 0.04
Sample size	1.0(1.0-1.0), 0.05	1.0(1.0-1.0), 0.05	1.0(1.0-1.0), 0.05	1.0(1.0-1.0), 0.06	1.0(1.0-1.0), 0.06	1.0(1.0-1.0), 0.07
Journal	1.1(0.8-1.6), 0.55	1.1(0.8-1.6), 0.54	1.1(0.8-1.6), 0.50	1.1(0.8-1.6), 0.57		
Impact factor	1.1(0.5-2.4), 0.84	1.1(0.5-2.4), 0.85				
Outcome	0.8(0.3-1.8), 0.58	0.8(0.3-1.8), 0.55	0.8(0.3-1.7), 0.54

**Table 7:** **Multivariable analysis of any use of clustering analysis after elimination of collinear variables, stepwise deletion for each specialist variable**

	**Model 1 OR(CI),** ***P*** **value**	**Model 2 OR(CI),** ***P*** **value**	**Model 3 OR(CI),** ***P*** **value**	**Model 4 OR(CI),** ***P*** **value**
Epidemiologist/Methodologist	3.3(1.0-10.6), 0.04	3.3(1.0-10.7), 0.04	3.3(1.0-10.5), 0.04	3.3(1.0-10.4), 0.04
Sample size	1.0(1.0-1.0), 0.06	1.0(1.0-1.0), 0.05	1.0(1.0-1.0), 0.06	1.0(1.0-1.0), 0.07
Journal	1.1(0.8-1.6), 0.50	1.1(0.8-1.6), 0.46	1.1(0.8-1.6), 0.55	
Impact factor	1.1(0.5-2.3), 0.89			
Outcome	0.8(0.3-1.7), 0.51	0.8(0.3-1.7), 0.50		
Biostatistician	1.5(0.6-4.1), 0.40	1.5(0.6-4.1), 0.40	1.5(0.6-4.0), 0.42	
Sample size	1.0(1.0-1.0), 0.03	1.0(1.0-1.0), 0.03	1.0(1.0-1.0), 0.04	1.0(1.0-1.0), 0.04
Journal	1.2(0.8-1.6), 0.36	1.2(0.8-1.7), 0.28	1.2(0.8-1.6), 0.34	1.2(0.9-1.5), 0.29
Impact factor	1.2(0.6-2.5), 0.61			
Outcome	0.8(0.4-1.7), 0.49	0.8(0.3-1.6), 0.48		
Any specialist	6.7(0.8-57.3), 0.08	6.9(0.8-58.8), 0.08	7.0(0.8-61.0), 0.08	7.0(0.8-60.7), 0.08
Sample size	1.0(1.0-1.0), 0.03	1.0(1.0-1.0), 0.04	1.0(1.0-1.0), 0.03	1.0(1.0-1.0), 0.04
Journal	1.2(0.8-1.6), 0.39	1.1(0.8-1.6), 0.44	1.2(0.8-1.6), 0.33	
Impact factor	1.2(0.6-2.7), 0.58	1.3(0.6-2.7), 0.55		
Outcome	0.8(0.4-1.8), 0.60			

## Discussion

Our study on the use of clustering analysis in orthopaedic research suggests that a small proportion of studies are currently employing these important statistical methods. Multivariable modeling of predictors associated with the presence of adjustment for clustering showed a strong and significant association between any type of clustering adjustment and inclusion of an epidemiologist/clinical trials methodologist on the study team.

Our study has several strengths and weaknesses. First, we systematically identified every RCT published in the top five journals of orthopaedic surgery between 2006 and 2010. This method of limiting to specific journals allowed for the entire target population of articles to be identified, as opposed to an electronic literature search that may miss potential articles meeting the inclusion criteria. Use of the top five journals also allows the assumption of a conservative estimate in our findings. But on the other hand, this cannot be generalized to other journals or to the broader orthopaedic literature. Also, while a single individual did inclusion for all articles, a second individual cross-checked a random selection of articles, which minimizes any selection bias.

Identified articles were then reviewed for inclusion and relevant data were extracted by a single researcher with experience in epidemiology and biostatistics. This extraction method allowed for consistency across articles and maintained homogenous definitions throughout the process; however, while there may be potential for bias due to extraction by a single reviewer, both authors met throughout the extraction process to clarify interpretations of extracted data. Despite efforts to extract all relevant data from all articles in the target population, data were underreported in several of the articles. Missing data were especially notable for the variables “biostatistician” and “epidemiologist/clinical trials methodologist”; the majority of author or study member specialties were not reported in the articles or easily identifiable from headings. In an effort to minimize the missing data, the corresponding author of each article was contacted and asked about the specialties of members of the study team. However, not all authors responded to the request for data. The underreporting here may bias our results. One possibility is that studies not reporting study member specialties may have been less likely to perform clustering analysis. If this was the case, our study would represent the higher-quality articles and therefore potentially be an over estimate of the use of clustering analysis. This hypothesis remains to be tested.

The method of stepwise regression used in the analysis of these data is controversial in some contexts, but generally remains an accepted method of hypothesis testing and generation. We are not aware of any other literature investigating predictors of accounting for clustering, and the investigational nature of this objective led us to this approach. Further studies are needed to verify these findings. Furthermore, the method of using GEEs for accounting for clustering in our analyses has recently been shown in Poisson data to increase the likelihood of type 1 errors [13], but not in binary outcomes. That is, in another paper Monte Carlo simulations showed that GEE models had better power at detecting within-cluster homogeneity than did other methods when examining binary outcomes [14]. We recommend additional simulations be carried out to determine the validity of this approach.

A final potential weakness of the study is the cut-off date of 2010. It is possible that in the year and a half between our cut-off date and the analysis of these data, levels of the use of clustering analysis in orthopaedic RCT studies have changed. However, there is no known identifiable event that would initiate such a change, making this a marginal concern. Overall, our analysis is only applicable to the year of papers we reviewed for these journals. But, we still hold that this analysis represents relatively recent RCTs in orthopaedic surgery and their use of clustering analyses.

Although several papers have previously demonstrated the importance of taking clustering into account in RCTs, this type of analysis has not yet become standard practice [7,10]. Our study suggests a low prevalence of adjustment for clustering effects in RCTs published in the orthopaedic literature, with only 21.5% of included articles using any of these important methods. To the best of our knowledge, our study is the first to look at potential predictors of the use of clustering adjustment in RCTs. Multivariable modeling of predictors associated with adjustment for clustering showed a strong and significant association between any type of clustering adjustment and inclusion of an epidemiologist/clinical trials methodologist on the study team. A large effect was also seen for the inclusion of any type of specialist (epidemiologist/clinical trials methodologist or biostatistician). This finding was expected, in that individuals specifically trained in clinical research methods are more likely to employ proper methodology. By demonstrating the association between an adjustment for clustering in a study and the presence of an epidemiologist or clinical trials methodologist on the study team, we are able to make recommendations for practical ways to improve the use of these important statistical methods. For example, the inclusion of an epidemiologist or clinical research methodologist in the study design phase *a priori* could ensure that proper methods are planned and implemented that limit or control for the effects of clustering (e.g., stratification, limiting the number of centers/providers, homogeneous cluster sizes, statistical analyses to adjust for clustering).

We were surprised to find that the inclusion of a biostatistician was not significantly associated with increased use of clustering adjustment methods. One potential explanation is that epidemiologists or clinical trial methodologists are often included from the design phase of a study, whereas biostatisticians are often only included in the analysis phase. Since our outcome is defined as accounting for clustering effects in either randomization or statistical analysis, involvement of a specialist *a priori* in the study is an important consideration. This *a priori* versus *ad hoc* inclusion may be associated with a greater use of proper adjustment techniques; however, this hypothesis remains to be tested.

In addition to a lack of proper author specialization on study teams, there are several other potential reasons that adjustment for clustering effects is not currently a common practice. As mentioned above, adjustment for clustering generally increases the sample size needed for a given power, making recruitment a longer or more difficult process and potentially increasing funding and other resource needs. This could act as a barrier to researchers who might initially be interested in examining clustering effects within their studies. We found that many of the included studies reported that the therapists had similar training or that there were no noted differences between therapists. But this is insufficient, as clustering effects may still exist and equality of therapists cannot be assumed. We recommend that clinical trialists perform these analyses where relevant and that institutional review boards and peer reviewers be careful to point out the need for these analyses. In addition, a set of standards could be developed that outline when and how these adjustments can be done, providing concrete examples and empirical evidence of this need.

The effect of clustering may be difficult to detect in studies that are underpowered when divided by cluster; however, statistical analyses that ignore the presence of potential clustering will most likely result in overly precise and therefore misleading estimates [1]. The methods for performing sample size calculations for studies with clustering effects depend on the type of data for the primary outcome of interest (e.g., continuous, binary, count). Several methods are suggested in the literature and several statistical packages have the ability to derive these estimates [15,16]. As an example, many studies use outcome measures that produce continuous data, for which an ICC is needed to calculate sample size; this requires an *a priori* knowledge of within- and between- cluster variances [17]. Several efforts are underway to encourage the use of clustering analysis through the creation of databases of ICCs for various outcomes used in surgical trials [1]. These databases will give researchers information on the likely magnitude of ICCs for different outcomes and enable the use of clustering effect estimates in the planning stages of a trial. This in turn will enable accurate sample size calculation in the design phase of a study and thus adequate power to test hypotheses [18]. Cook et al. [1] suggest that the optimal use of available data would involve a formal meta-analysis of ICC estimates. Furthermore, more work is needed on sample size calculations and methods of accounting for clustering for binary and count data in clinical research. This important research should be prioritized, with the goal of informing researchers of possible clustering effects by outcome and enabling better practices in analyses through *a priori* understanding of potential clustering effects.

## Conclusions

On the basis of our findings, we see a need for the improvement in methodology when dealing with clustering in RCTs. Strongly associated with adjusting for clustering was the inclusion on the study team of a specialist in biostatistics and/or epidemiology/clinical trials methodology. Investigators planning RCTs should make careful selection of their study teams to ensure that proper expertise is included. Additionally, the use of databases categorizing ICCs for different outcomes from the planning stages of a trial will improve sampling and study design and help reduce the effects of clustering.

## References

[1] Cook JA, Bruckner T, MacLennan GS, Seiler CM (2012). Clustering in surgical trials–database of intracluster correlations. Trials.

[2] Roberts C, Roberts SA (2005). Design and analysis of clinical trials with clustering effects due to treatment. Clin Trials.

[3] Roberts C (1999). The implications of variation in outcome between health professionals for the design and analysis of randomized controlled trials. Stat Med.

[4] Walwyn R, Roberts C (2010). Therapist variation within randomised trials of psychotherapy: implications for precision, internal and external validity. Stat Methods Med Res.

[5] Cook JA, Ramsay CR, Fayers P (2004). Statistical evaluation of learning curve effects in surgical trials. Clin Trials.

[6] Walters SJ (2010). Therapist effects in randomised controlled trials: what to do about them. J Clin Nursing.

[7] Lee KJ, Thompson SG (2005). The use of random effects models to allow for clustering in individually randomized trials. Clin Trials.

[8] Biau DJ, Halm JA, Ahmadieh H, Capello WN, Jeekel J, Boutron I (2008). Provider and center effect in multicenter randomized controlled trials of surgical specialties: an analysis on patient-level data. Ann Surg.

[9] Chu R, Thabane L, Ma J, Holbrook A, Pullenayegum E, Devereaux PJ (2011). Comparing methods to estimate treatment effects on a continuous outcome in multicenter randomized controlled trials: a simulation study. BMC Med Res Method.

[10] Biau DJ, Porcher R, Boutron I (2008). The account for provider and center effects in multicenter interventional and surgical randomized controlled trials is in need of improvement: a review. J Clin Epidemiol.

[11] Thompson Reuters Web of Science (2010). Journal citation reports.

[12] Peduzzi P, Concato J, Kemper E, Holford TR, Feinstein AR (1996). A simulation study of the number of events per variable in logistic regression analyses. J Clin Epidemiolo.

[13] Gao D, Grunwald GK, Xu S (2013). Statistical methods for estimating within-cluster effects for clustered poisson data. J Biomet Biostat.

[14] Austin PC (2010). A comparison of the statistical power of different methods for the analysis of repeated cross-sectional cluster randomization trials with binary outcomes. Int J Biostat.

[15] Hemming K, Girling AJ, Sitch AJ, Marsh J, Lilford RJ (2011). Sample size calculations for cluster randomized controlled trials with a fixed number of clusters. BMC Med Res Method.

[16] Reich NG, Myers JA, Obeng D, Milstone AM, Perl TM (2012). Empirical power and sample size calculations for cluster-randomized and cluster-randomized crossover studies. PLoS One.

[17] Killip S, Mahfoud Z, Pearce K (2004). What is an intracluster correlation coefficient? Crucial concepts for primary care researchers. Ann Fam Med.

[18] Preisser JS, Reboussin BA, Song E-Y, Wolfson M (2007). The importance and role of intracluster correlations in planning cluster trials. Epidemiology.

